# First person – Lelei Wen

**DOI:** 10.1242/bio.057521

**Published:** 2020-11-12

## Abstract

First Person is a series of interviews with the first authors of a selection of papers published in Biology Open, helping early-career researchers promote themselves alongside their papers. Lelei Wen is first author on ‘Influence of maternal diet on offspring survivorship, growth, and reproduction in a sheetweb spider’, published in BiO. Lelei is a PhD student in the lab of Daiqin Li at Hubei University, Wuhan, China, investigating spider nutrition ecology.


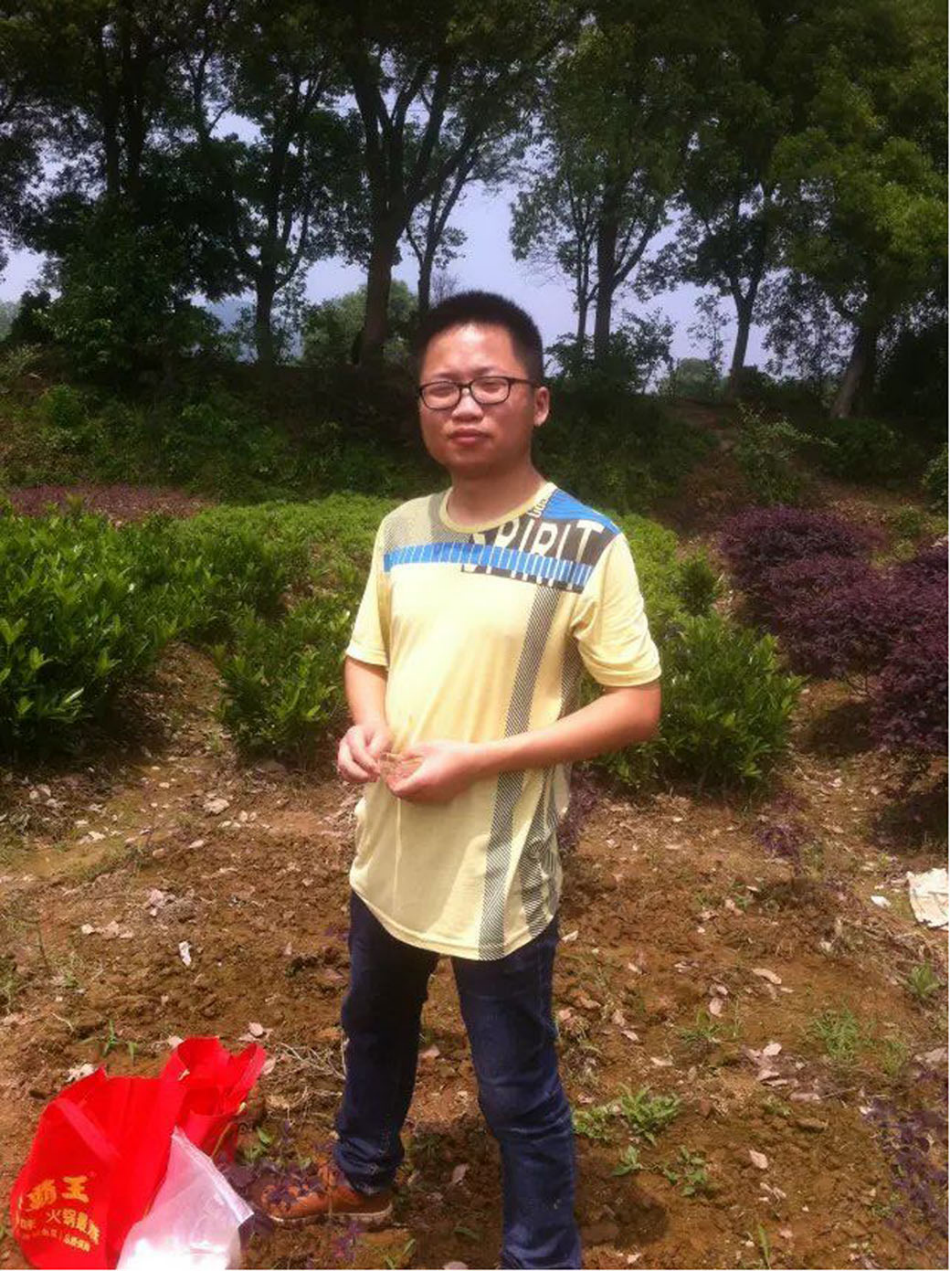


**Lelei Wen**

**What is your scientific background and the general focus of your lab?**

General research interests of our lab lie in the fields of ecology, behaviour and evolution of animals, mostly in terrestrial arthropods, particularly spiders.

**How would you explain the main findings of your paper to non-scientific family and friends?**

Pregnant mother spiders that eat nutritious prey have healthy babies.

**What are the potential implications of these results for your field of research?**

We should consider the potential maternal effect when studying spider nutrition.

**What has surprised you the most while conducting your research?**

A single meal immediately before oviposition is sufficient to influence offspring survival, growth, and daughters' reproduction in the sheetweb spider *Hylyphantes graminicola*.

**Figure:**
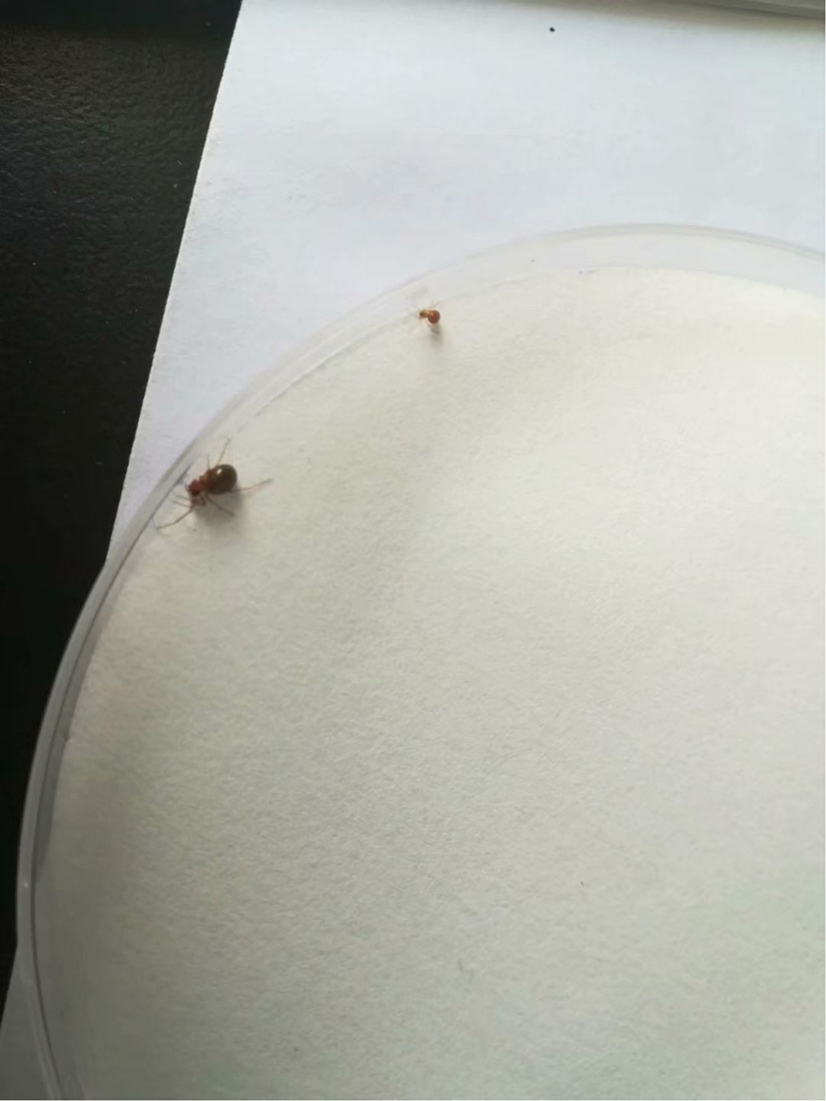
**Juvenile spiders fed together but one is mature and one remains small.**

**What, in your opinion, are some of the greatest achievements in your field and how has this influenced your research?**

Mayntz and Toft (2001) was the first to culture fruit flies on normal medium supplemented with various nutrients (e.g. amino acids and fatty acids). This and subsequent studies demonstrated that altering the macronutrient content of the medium on which larval fruit flies develop induces changes in the macronutrient contents of the adult flies. These changes then influence the survival, growth and reproduction of spiders that feed on the adult flies. We followed their method to study on how macronutrients or micronutrients affect spider performance.

**What's next for you?**

I am trying to explain why spiders usually have moulting problems when fed in the lab.
